# Ploidy plasticity drives fungal resistance to azoles used in agriculture and clinics

**DOI:** 10.1371/journal.pbio.3003083

**Published:** 2025-04-02

**Authors:** Kaustuv Sanyal, Aswathy Narayanan

**Affiliations:** 1 Molecular Mycology Laboratory, Molecular Biology and Genetics Unit, Jawaharlal Nehru Centre for Advanced Scientific Research, Bangalore, India; 2 Department of Biological Sciences, Bose Institute, Unified Academic Campus, Bidhannagar, Kolkata, India

## Abstract

The rapid growth in antimicrobial resistance is of great medical concern. This Primer highlights a new study in PLOS Biology that unveils the link between ploidy plasticity and the emergence of antifungal resistance in *Candida tropicalis*.

## Teaser

The rapid growth in antimicrobial resistance is of great medical concern. A new study in *PLOS Biology* unveils the link between ploidy plasticity and the emergence of antifungal resistance in *Candida tropicalis.*

The fungal kingdom encompasses species with a wide variety of habitats: some thrive as free-living organisms, while others are commensals residing in host systems. A few commensals can become opportunistic pathogens once the host’s immune system is compromised. Among this vast, heterogeneous group of fungal species, there are some that are found in the environment, causing infections in humans and other animals. The impact of fungal pathogens in healthcare and economy has been overlooked for decades, but we are beginning to comprehend the potential effects of anthropogenic activities on the fungal kingdom in the environment [1]. Factors such as the rampant use of high doses of antifungals in poultry and fungicides in agricultural farmlands combined with global warming are thought to be contributing to an alarming increase in drug resistance and the emergence of new pathogens. Azoles are widely used antifungals in clinics; they are also the major component of agricultural fungicides used globally [1]. How do azole-based fungicides used in agriculture lead to the emergence of drug-resistant strains that exhibit cross-resistance to clinical azoles? A recent study in *PLOS Biology* by Hu and colleagues provides insights into novel mechanisms underlying the acquired resistance in *Candida tropicalis*.

*C. tropicalis*, an opportunistic fungal pathogen, belongs to the phylum Ascomycota. *C. tropicalis* is also isolated from environmental niches—its natural habitats include environmental reservoirs like forest soil, agricultural fields and water bodies [2]. However, it is also classified under high-priority fungal pathogens by the World Health Organization and is a major pathogen in tropical regions, causing superficial and invasive infections. *C. tropicalis* invasive infections are associated with a mortality rate of 55%–60% and the pathogen exhibits high azole resistance, a growing medical concern [3]. Previous studies indicate that exposure to agricultural azoles in natural environments can facilitate the emergence of clinical azole resistance in *C. tropicalis* [4,5].

**Fig 1:**
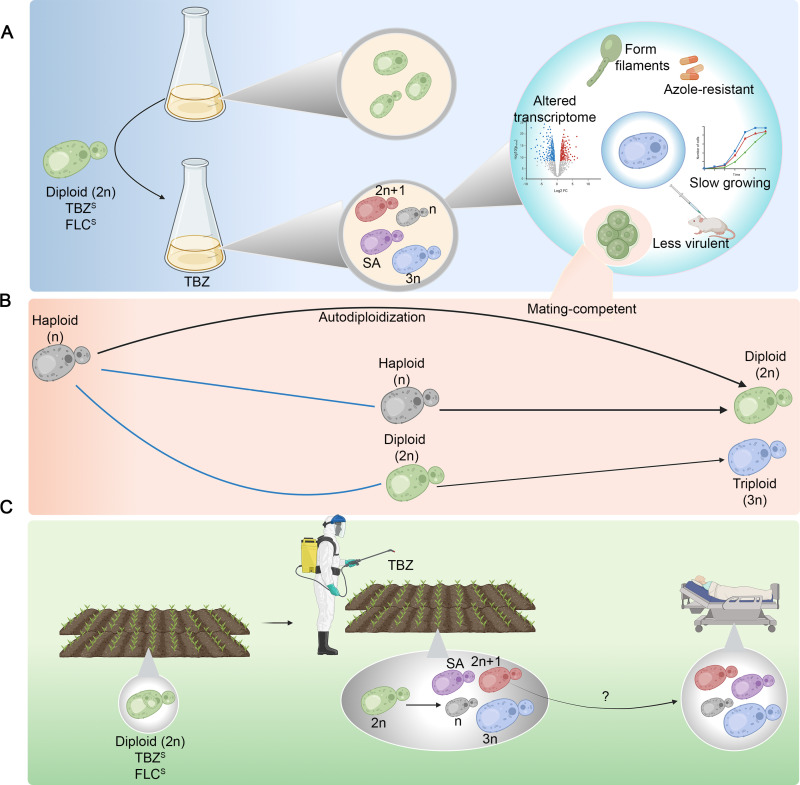
Tebuconazole-induced ploidy plasticity and azole resistance in *Candida tropicalis.* (**A**) On experimentally evolving drug-susceptible, diploid (2n) *C. tropicalis* strains in the presence of an agricultural fungicide tebuconazole (TBZ), cells of varying ploidy states can be obtained including haploid (n), triploid (3n), aneuploid (such as 2n + 1) and segmental aneuploid (SA) cells. Owing to the ploidy shifts and an altered transcriptome, haploid cells exhibit resistance to both agricultural and clinical azoles, are mating-competent, and undergo filamentation—all at a fitness cost reflected in the growth rate and virulence. (**B)** Mating observed in haploid cells is shown. Mating ploidy states are connected by blue lines, and the resulting ploidy states by black arrows. (**C**) Model depicting the emergence of azole-resistant strains in the farmlands, cross-resistance to clinical azoles and possible transmission to clinics. Created using Biorender.com.

A classical approach to studying the emergence of antifungal drug resistance is experimental evolution in which a drug-susceptible strain is grown in the presence of the drug under controlled laboratory conditions. Hu *and colleagues* employ an experimental evolution strategy in which azole-susceptible *C. tropicalis* strains are exposed to incrementally increasing concentrations of tebuconazole (TBZ), an azole-based fungicide used widely in agriculture (Fig 1A). Interestingly, the TBZ-resistant colonies obtained in the experimental evolution regime exhibit cross-resistance to the clinical azoles, too, [6] confirming the possibility of azole-resistant strains emerging in environmental niches.

*Candida albicans and C. tropicalis,* two abundant fungal pathogens, occur in a diploid (2n) state. Both species are primarily asexual fungi and are known to undergo parasexual mating, in which two diploid cells of opposite mating types fuse to generate tetraploid (4n) cells. The resulting cells undergo concerted chromosome loss, generating cells of various ploidy states [7,8]. However, the most common naturally occurring ploidy state in *C. tropicalis* is diploid. Interestingly, some TBZ-resistant colonies obtained are haploid (n), a previously unknown ploidy state for this organism, indicating that agricultural azoles can induce ploidy shifts in the *C. tropicalis* isolates.

Even *C. albicans* can exist in a mating-competent haploid state that is unstable [9]. Is haploidy a less stable state in *C. tropicalis* too? The haploid cells obtained by Hu and colleagues could mate with cells of different ploidy states (Fig 1B). Haploid strains of opposite mating types gave rise to diploid cells on mating. Haploid cells could even efficiently mate with diploid progenitors to yield triploid (3n) cells. This versatility, combined with the autodiploidization (n becomes 2n) observed in a fraction of haploid cells, raises the possibility that haploidy is a transition state in *C. tropicalis*. It is tempting to assume that frequent hybridization processes are facilitated by multiple ploidy states in an environmental niche. Indeed, potential hybrids of *C. tropicalis* were previously isolated from environmental sources [10]. The study by Hu *and colleagues* also reveals that haploid cells grow slower and are less virulent in a mouse model of systemic infection, indicating a reduced fitness compared to the diploid strains. The authors report the existence of haploid *C. tropicalis* clinical isolates by analyzing publicly available whole genome sequencing data revealing these isolates possess low heterozygosity in the genomic DNA, similar to the haploids obtained in the experimental evolution regime.

Besides ploidy plasticity, the authors find copy number variations in azole resistance-related genes like *TAC1* and *ERG11*. *TAC1* is a transcriptional regulator of drug-efflux pumps, and *ERG* genes are involved in the biosynthesis of ergosterol, a major fungal cell membrane component. In *C. albicans,* a closely related species of *C. tropicalis*, an isochromosome with the duplicated short arm of chromosome 5 bearing additional copies of *ERG11* and *TAC1* confers azole resistance [11]. Copy number variations of *ERG11* has been implicated previously in azole resistance in multiple fungal pathogens including *C. tropicalis* [4,12,13]. The study of Hu and colleagues raises the possibility that isochromosome formation could be a general mechanism operating across species, triggered by clinical and agricultural azoles (Fig 1C). How stable are the isochromosomes if at all formed in *C. tropicalis*? Continuous passages in the absence of the drug can reveal the role of drug pressure in the maintenance of these additional copies of genes conferring drug resistance.

While the fully assembled genome, including haplotypes, of a diploid *C. tropicalis* strain is available [14], many aspects of the pathobiology of this fungal pathogen remain largely unexplored. The availability of a stable haploid state will facilitate gene function studies, including the importance of recessive alleles. In addition, studying changes in the proteome in a haploid compared to the diploids will help better understand the physiological impact of ploidy shifts. Haploidy and the ability of haploid cells to mate to produce various ploidy states may prove to be the arsenal of *C. tropicalis* to be one of the most successful drug-resistant fungal pathogens.
